# Ocrelizumab-induced colitis—critical review and case series from a Romanian cohort of MS patients

**DOI:** 10.3389/fneur.2025.1530438

**Published:** 2025-02-05

**Authors:** Ileana Maria Vodă, Vlad Eugen Tiu, Luiza Răuță, Paul Ciucur, Andreea Ioana Mușuroi, Alina Flavia Tomescu, Nicoleta Laura Humă, Florin Dobrițoiu, Elena Terecoasă, Lucian Negreanu, Cristina Tiu

**Affiliations:** ^1^Bucharest University Emergency Hospital, Bucharest, Romania; ^2^Elias University Emergency Hospital, Bucharest, Romania; ^3^Clinical Neurosciences, Carol Davila University of Medicine and Pharmacy, Bucharest, Romania; ^4^Internal Medicine, Carol Davila University of Medicine and Pharmacy, Bucharest, Romania; ^5^Pathoteam Diagnostic Laboratory, Bucharest, Romania

**Keywords:** multiple sclerosis, drug induced colitis, colitis, ocrelizumab, review, CD-20

## Abstract

**Background:**

Widespread use of ocrelizumab, an anti-CD20 monoclonal antibody, for treating patients with multiple sclerosis (MS), has led to an increase in reported adverse events following real-world observation. Among these, drug-induced colitis is a rare, but severe side effect, prompting a recent FDA statement regarding this safety concern. Objectives: We analyzed a cohort of ocrelizumab treated patients in our MS center to evaluate the incidence of drug-induced colitis.

**Methods:**

We present a critical review of the available literature on diagnosis and management of anti-CD20 induced colitis and display a case series of 3 suspected patients in our cohort.

**Results:**

Two patients met the full criteria for ocrelizumab-induced colitis, while a third partially met the criteria. Following symptomatic treatment and discontinuation of ocrelizumab, the patients showed favorable outcomes.

**Conclusion:**

Ocrelizumab-induced colitis is a rare, but severe adverse event. Its incidence may be higher than expected, reaching 1,95% in our cohort of MS patients. Further reporting of such cases is essential to broaden our understanding of this side effect.

## Introduction

Ocrelizumab is an anti-CD20 humanized IgG1 monoclonal antibody used as a high-efficacy disease-modifying treatment (DMT) for relapsing–remitting multiple sclerosis (RRMS) and so far the only therapy approved for primary-progressive multiple sclerosis (PPMS) (1–4). Ocrelizumab is proven to be less immunogenic than its predecessor, rituximab, an anti-CD20 chimeric antibody (5). The primary safety reports for ocrelizumab list infusion-related reactions and infections (mainly upper respiratory tract infections) as the most common adverse reactions, however there are rare cases of severe adverse events that have been reported, such as malignancies or progressive multifocal leukoencephalopathy (PML) (1, 3, 5). Ocrelizumab-induced colitis is a rare occurrence, with only a few cases being reported in literature so far (6–15). We present three cases of ocrelizumab-induced colitis in a cohort of patients with relapsing–remitting multiple sclerosis treated with ocrelizumab in our clinic.

## Methods

### Study design and participants

We conducted a monocentric, observational, cross-sectional study on a cohort of patients from our MS center that were treated with ocrelizumab.

### Eligibility criteria

- Age > 18 years
- Diagnosis of MS (RRMS or PPMS)
- Current treatment with or a history of use of ocrelizumab in the last 12 months
- Digestive symptoms or signs (e.g., diarrhea, weight loss, bloody stools, abdominal cramps or pain) with an onset after initiation of anti-CD20 therapy

### Study variables and protocol

A questionnaire was administered to all the ocrelizumab-treated subjects from our MS center. The questionnaire addressed the presence of any digestive symptoms and signs, date of onset, frequency and evolution of symptoms, current treatment and response to this treatment, whether infectious screening was already performed, whether there was an available gastrointestinal evaluation (survey available on request to corresponding author). The purpose of this survey was to highlight patients under ocrelizumab therapy that had new-onset digestive symptoms.

### Outcome measures

The aim of the study was to determine the incidence and severity of digestive symptoms in patients with MS (pwMS) that were being treated or had a recent history of treatment with ocrelizumab.

### Statistical analysis

The data was segregated by responses and each item was assessed using Microsoft Excel (Seattle, United States). Demographic and baseline characteristics were described using medians and interquartile range or means and standard deviation for continuous variables, depending on the normality of the distribution, and percentages for categorical data.

### Ethics

Informed consent was obtained from each participant prior to their inclusion in this study. The study protocol was approved by the local ethics board prior to its commencement.

### Diagnostic criteria for ocrelizumab-induced colitis

For the diagnosis of ocrelizumab-induced colitis, the diagnostic criteria proposed by Quesada-Simó et al. (16) were applied. The diagnosis of ocrelizumab-induced colitis was made if at least three major criteria and two minor ones were met:

- Major diagnostic criteria:
- Exposure to an anti-CD20 drug in the previous year.
- Compatible symptoms that may include fever, abdominal pain, watery or muco-bloody diarrhea.
- CD20+ cell depletion on gastrointestinal (GI) biopsy.
- Lymphoplasmocytic infiltrate (CD3+ and CD79+ plasma cells) in the lamina propria.
- Clinical/ endoscopic recovery after drug withdrawal and CD20+ cell recovery in intestinal mucosa.
- Minor diagnostic criteria:
- Elevation of acute inflammation biomarkers in laboratory tests (C-reactive protein - CPR, erythrocyte sedimentation rate - ESR, lymphocytosis).
- Compatible endoscopic findings (mucosal erythema with edema and patchy ulcer/ erosions with predominant involvement of the ileum and proximal colon; tendency to spare stomach and duodenum).
- Chronic active inflammation with cryptitis, goblet cell reduction, and superficial ulcers with areas of spared mucosa.
- Good response to glucocorticoid therapy.
- Absence of other possible etiologies:
- Normal neutrophil count.
- Absence of infectious causes (Cytomegalovirus - CMV, *Salmonella*, *Shigella*, *Campylobacter*, *Escherichia coli*, *Clostridium difficile*).
- Other comorbidities that could justify the pathology.
- Other drugs/ toxics that could justify the pathology.

## Results

The baseline cohort included 154 eligible patients with MS (pwMS) from our clinic, the MS center of the University Emergency Hospital of Bucharest. Of these, 143 (92,86%) were currently treated with ocrelizumab, while 11 patients (7,14%) had a history of ocrelizumab therapy in the last 12 months. For two out of this last category of patients, the withdrawal from anti-CD20 therapy was decided due to digestive symptoms which were interpreted by the attending physicians as drug-induced colitis (such as abdominal pain, abdominal cramps, diarrhea, bloody or watery stools). Out of the 143 patients currently treated with ocrelizumab, one patient reported new-onset digestive symptoms (Case 3).

Our survey identified three (1,95%) potential cases of digestive symptoms induced by ocrelizumab, out of the 154 patients who were treated with or had a recent history of ocrelizumab treatment. Two cases met the diagnostic criteria for ocrelizumab-induced colitis, while the third one fulfilled them only partially. A favorable outcome was achieved through symptomatic treatment and cessation of ocrelizumab for the first two cases, while the third is currently under evaluation. The key features of these cases were: a long period of administration of the anti-CD20 drug until onset of symptoms for the first case, overlapping infectious pathology which led to a delayed diagnosis for the second one and a scarce clinical picture in contrast to the presence of histopathological changes for the third.

A summary of the main characteristics of the selected patients can be found in Appendix 1.

### Case 1

A 35-year-old female patient was diagnosed with aggressive RRMS in 2021 and ocrelizumab was initiated in April 2021, with no initial side effects, no new relapses and no new lesions on follow-up MRIs. At the end of August 2023, after 6 infusions of ocrelizumab, the patient reported the onset of abdominal cramps, followed by diarrhea (7–9 stools/ day). These symptoms led to important weight loss (approximately 4 kg). With this being the first manifestation since initiation of ocrelizumab, no relationship to the administration of succeeding infusions could be established.

Her routine laboratory tests (September 2023) showed biological inflammatory syndrome and an elevated level of fecal calprotectin (5,356 mcg/ g). The lymphocyte subset count showed low levels of CD19+ B cells (1%) and NK lymphocytes (4%), and the immunogram revealed hypogammaglobulinemia (low IgG – 588 mg/dL, IgM and IgA within normal range). Infectious screening was performed (stool culture, stool ova and parasite test, *C. difficile* testing) with negative results for bacterial or parasitic infections. Empiric antibiotic therapy and anti-inflammatory treatment (local and systemic) showed no clinical benefit. An abdominal CT scan revealed minimal edema of the transverse, descending and sigmoid colon.

**Figure 1:**
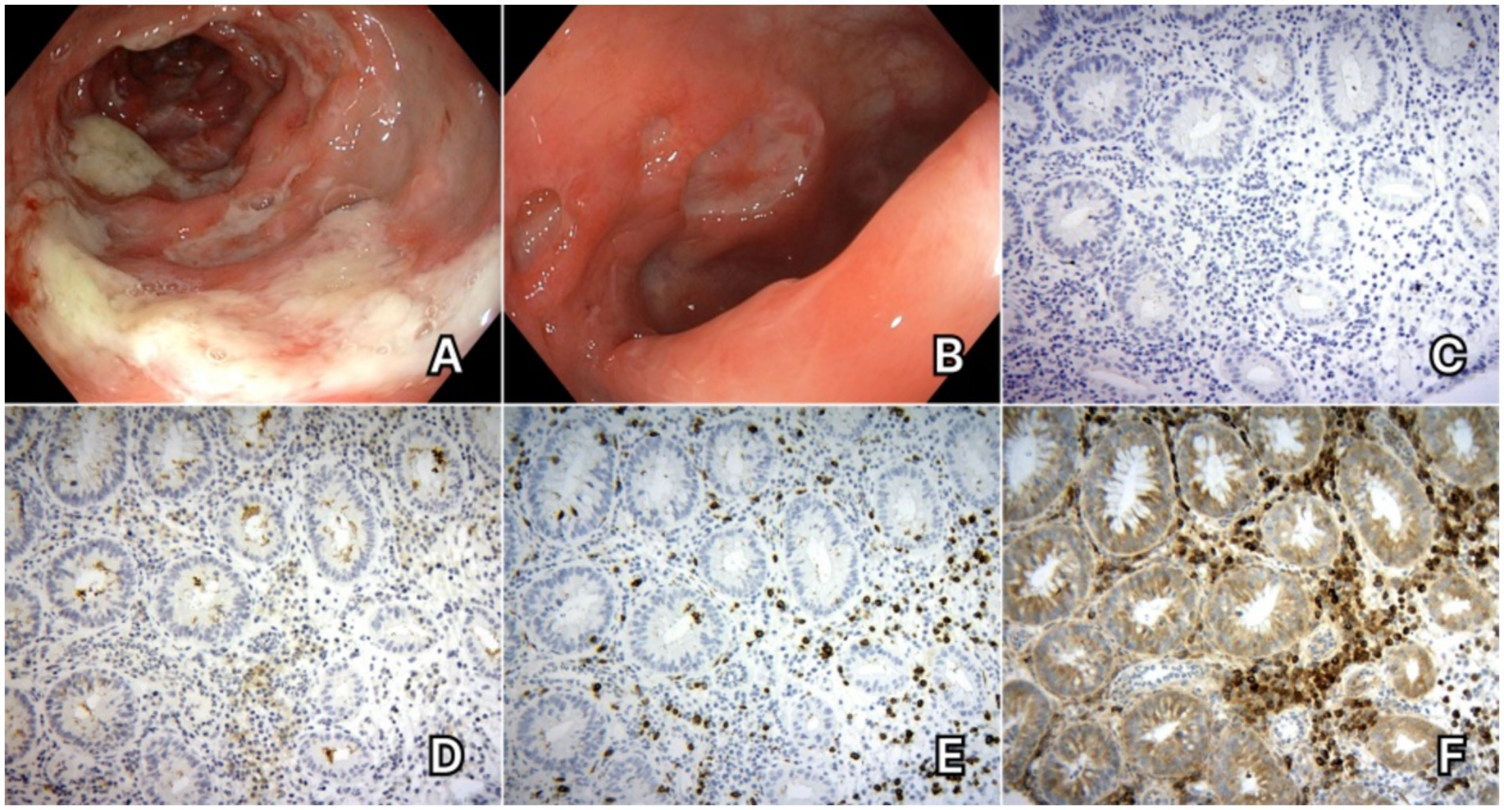
Case 1. **(A)** Colonoscopy aspects - multiple profound ulcerations which become confluent. **(B)** Colonoscopy aspects – profound ulcerations of the colonic mucosa. **(C)** Immunohistochemical staining for CD20 – absence of CD20+ B cells in the colonic mucosa. **(D)** Immunohistochemical staining for CD19 – absence of CD19+ B cells in the colonic mucosa. **(E)** Immunohistochemical staining for CD3 – CD3+ T cells are present in the colonic mucosa. **(F)** Immunohistochemical staining for CD79 – CD79a + B cells can be observed in the colonic mucosa.

Colonoscopy examination showed multiple profound ulcerations of the sigmoid and descending colon, which became confluent at the level of the transverse colon (Figures 1A,B). Biopsies demonstrated a diagnosis of focal active colitis, showing regenerative areas, microabscesses and erosions, without signs of intraepithelial dysplasia or neoplastic proliferation. The immunohistochemistry results revealed complete (severe) depletion of CD19+ and CD20+ lymphocytes (Figures 1C,D), with CD79a + B cells and CD3+ T cells being present (Figures 1E,F). Immunohistochemical staining for CMV was negative.

Extensive testing ruled out an infectious cause or inflammatory bowel disease (IBD). When applying the proposed criteria for ocrelizumab-induced colitis, our first patient met 3 major and 4 minor ones (Appendix 2), and a diagnosis of ocrelizumab-induced colitis was established.

She was started on intravenous methylprednisolone (IVMP) and mesalamine with a quick favorable response to therapy and a full resolution of symptoms in the following month. The patient was discharged with a 7-week oral corticosteroid tapering, with monthly neurological examination and close gastroenterological surveillance, and ocrelizumab treatment was stopped. Natalizumab was initiated 3 months later.

Follow-up colonoscopy at 3 months showed favorable evolution, with scar tissue and inflammatory pseudopolips of the transverse and sigmoid colon and millimetric erosions of the ascending colon. Immunohistochemistry staining showed repopulation of CD20+ B cells in the lamina propria of the colon.

### Case 2

A 26-year-old patient was diagnosed with RRMS in October 2019. After being initially treated with Glatiramer acetate for 18 months, he was switched to ocrelizumab in March 2022 due to accumulation of new lesions on follow-up MRIs. 1 month after initiation of anti-CD20 treatment, the patient presented digestive symptoms, with abdominal pain, cramps and diarrhea. After subsequent infusions, he noticed aggravation of the symptomatology shortly after administration of ocrelizumab, with progressive, yet incomplete, alleviation during the interval between infusions. The patient did not report these symptoms to the MS center.

In July 2023, following a course of antibiotic therapy after a dental extraction, the patient experienced aggravated symptoms (abdominal pain, bloody stools, weight loss). His laboratory results indicated elevated levels of fecal calprotectin. Initial investigations led to a diagnosis of *Clostridium difficile* enterocolitis. Symptoms continued despite treatment and resolution of *C. difficile* toxin. Follow-up testing for these symptoms was later interpreted as a second infection with *Campylobacter jejuni* after a positive test in October 2023.

**Figure 2:**
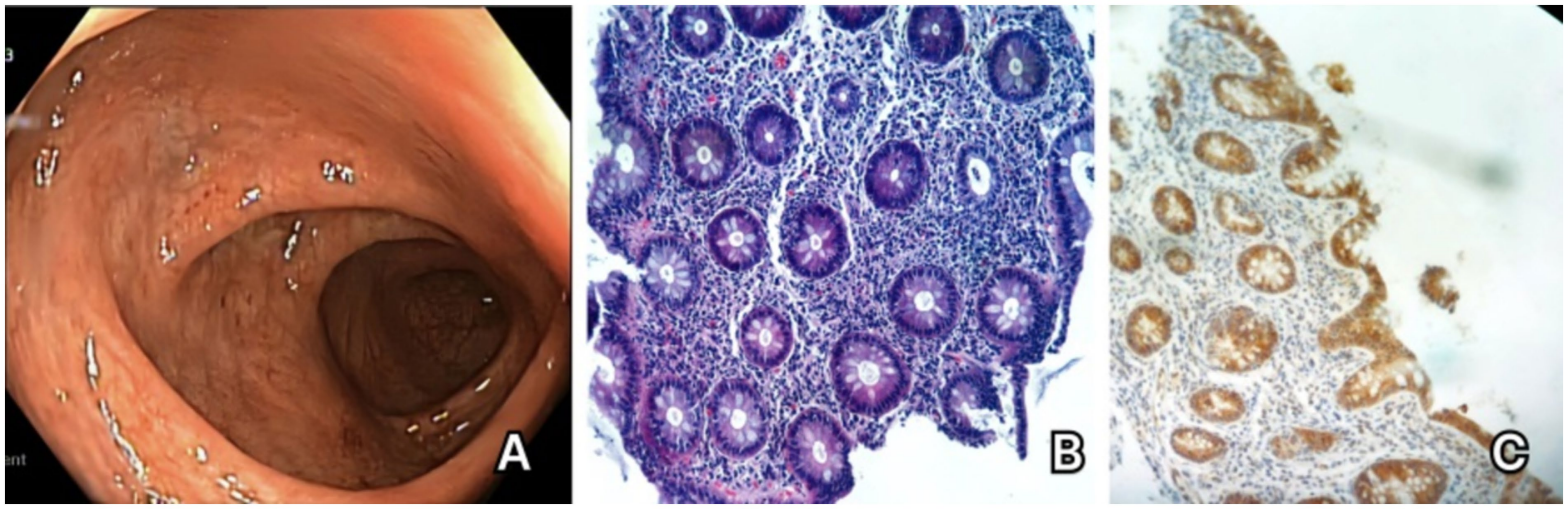
Case 2. **(A)** Colonoscopy showing erythema of the descending and sigmoid colon. **(B)** Hematoxylin and eosin staining of the colonic mucosa – important lesions of cryptitis. **(C)** CD20 immunohistochemical staining – absence of CD20 + cells in the colonic mucosa.

Due to the persistence of digestive symptoms for over 8 months and after ruling out a relapse of infectious enterocolitis via repeated microbial tests with negative results, a colonoscopy was performed in November 2023, which revealed erythematous areas of the transverse colon and rare microerosions with edema and erythema of the descending and sigmoid colon and the rectum (Figure 2A). Chronic active non-granulomatous colitis was detected on the histopathological examination (Figure 2B) and the immunohistochemical staining showed near-total depletion of CD20+ B cells (Figure 2C) with abundant CD79a + cells (lymphocytes and plasma cells) and relatively frequent CD3+ T cells in the lamina propria of the colon. Based on the analyzed biopsy, the diagnosis of active focal colitis (most probably drug-induced) was established.

The patient was started on anti-inflammatory treatment with mesalamine and oral corticosteroids, with favorable evolution. Ocrelizumab was discontinued, with progressive remission of the digestive symptoms during the following 6 months, and the patient is currently being evaluated for initiation of cladribine due to high JCV antibody index.

While the patient fulfilled all the major and minor proposed diagnostic criteria for drug-induced colitis (Appendix 2), the superposed infections represented important exclusion criteria. However, the timely association between symptom onset and anti-CD20 therapy initiation and the careful reevaluation of the patient’s history proved that manifestations which were highly suggestive for a drug-induced adverse event have been present for a longer period before their exacerbation by infections.

### Case 3

A 37-year-old patient diagnosed with RRMS in 2022. She was considered to have an aggressive form of MS and was started on ocrelizumab in March 2022. Initial clinical evolution was favorable, with no new relapses or side effects. In April 2024, the patient reported episodes of watery stools (1–2 per day, with each episode lasting 1 week, followed by a symptom-free period of 1 month), sometimes accompanied by blood streaks, with an onset approximately 1 year prior and without apparent correlation to the timing of infusions. No other digestive symptoms were present. No biologic abnormalities were found, the fecal calprotectin level was within normal limits and infectious screening was negative.

**Figure 3:**
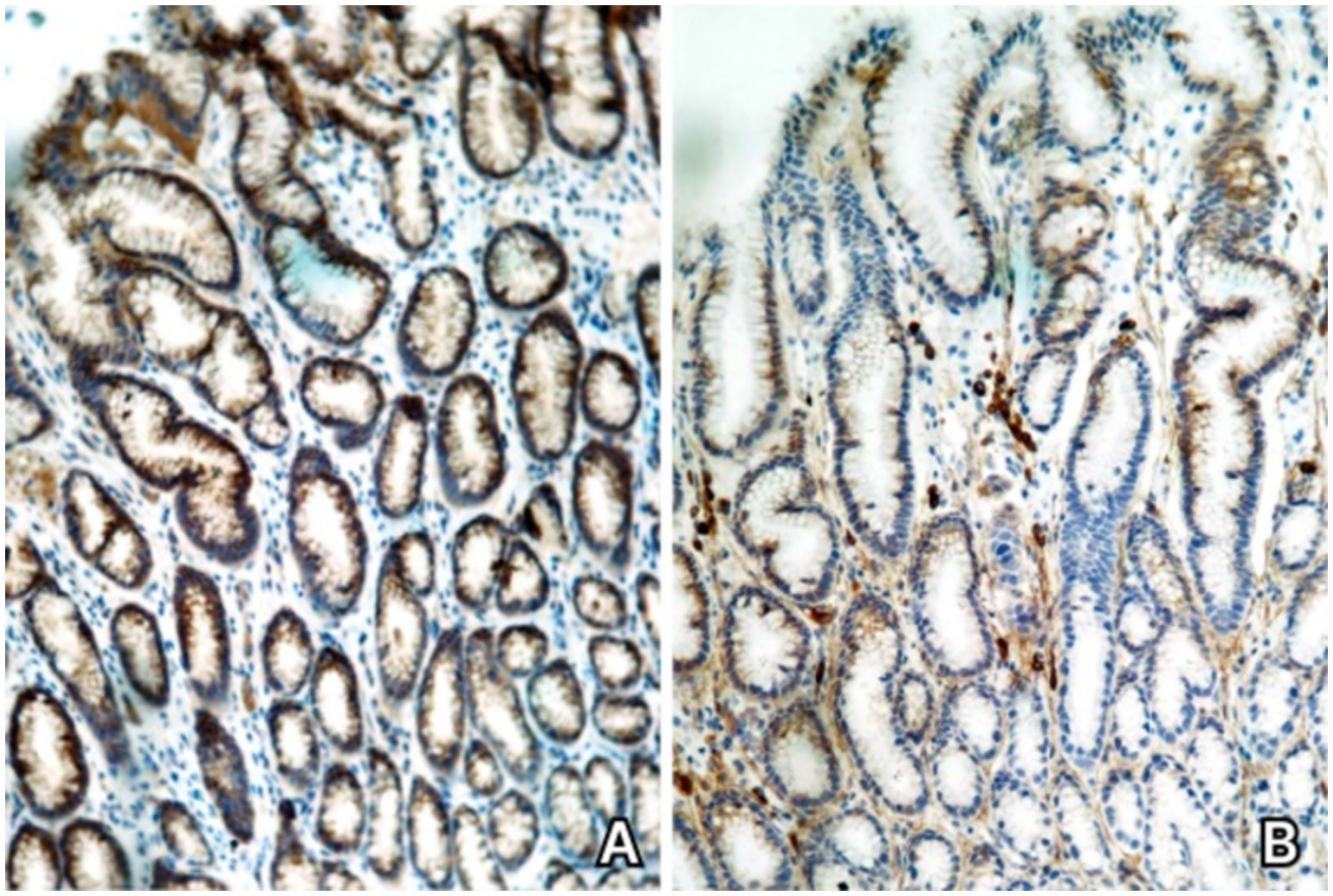
Case 3. **(A)** Immunohistochemical staining for CD20 – absence of CD20+ B cells in the gastric mucosa. **(B)** Immunohistochemical staining for CD79 – CD79+ B cells are present in the gastric mucosa.

The patient underwent an upper and lower GI tract endoscopy, yet no macroscopic abnormalities were found. Histopathological results did not reveal signs of inflammation or dysplasia. Immunohistochemical staining showed the absence of CD20+ B cells in the lamina propria of the gastric mucosa (Figure 3A), with CD79a + B cells and CD3+ T cells being present (Figure 3B), findings which were attributed to chronic immunomodulatory treatment with ocrelizumab. In the absence of active inflammation, a diagnosis of colitis could not be established.

When applying the proposed diagnostic criteria, 3 major ones but none of the minor criteria are met (Appendix 2). Without paraclinical and histopathological arguments attesting inflammation, no diagnosis of colitis can be established following current guidelines. However, given the timely association to the onset of anti-CD20 therapy and the clinical and histopathological findings, this case is considered to be an incipient form of ocrelizumab-induced colitis, even in the absence of minor diagnostic criteria. A follow-up for a longer period of time is warranted in order to accurately report the patient’s evolution.

## Discussion

### Ocrelizumab-induced colitis – pathophysiological aspects and potential mechanisms

Multiple sclerosis (MS) is a chronic, autoimmune, demyelinating disease that affects the central nervous system (CNS). Although MS was mainly considered to be a T cell mediated pathology, recent studies underline the role of B cells in its pathophysiology (1, 2, 17). Interestingly, B cells have different phenotypes, and a notable subtype is that of regulatory B cells (Breg), which decrease a potentially harmful and excessive inflammatory response (18). Bregs secrete IL-10, an anti-inflammatory cytokine, which acts to inhibit the inflammatory T cell response and the release of pro-inflammatory cytokines while suppressing cellular immune responses (19–22). It is important to mention that Breg function is altered in MS patients (18) - *in vitro* studies have shown that B cells secrete mainly pro-inflammatory molecules and lower levels of regulatory ones (17, 20).

Ocrelizumab is a humanized monoclonal IgG1 antibody which leads to depletion of CD20-positive cells (3, 18). Anti-CD20 therapies act to inhibit certain types of B cells, leaving pro-B cells, plasmablasts and plasma cells unaffected, which in turn leaves immunoglobulin production and repopulation of B cells unaltered (3, 5, 17). Once the lymphocytic repopulation takes place, B cells exhibit a modified phenotype, secreting less pro-inflammatory cytokines and higher levels of anti-inflammatory ones, which can be regarded as a form of immune reconstitution (3, 5, 17). The repletion of B cells consists mostly of immature or naïve cells, with ongoing suppression of memory B cells up to 2 years after withdrawal of anti-CD20 therapy (17, 20, 23).

Ocrelizumab-induced colitis is a rare, but potentially severe adverse reaction, with 39 cases being reported in literature and in the FAERS (Food and Drug Administration Adverse Event Reporting System) database, although only three cases had a probable causal association and the other 35 cases had a possible causable association with this monoclonal antibody therapy as per Kim et al. (7–15, 24, 25). The FAERS report was stated in August 2022. Other 12 cases were described in literature, excluding three cases of diverticulitis (6, 10, 26–31). This adverse event appears to be more frequent for patients under rituximab therapy (32–46), which might be explained by: 1. A longer and more extensive history of use for rituximab compared to ocrelizumab (16, 47). 2. Use of rituximab in association with other immunosuppressants in rheumatological or hematological diseases, which leads to cumulative adverse events (16); 3. The chimeric structure of rituximab, which makes it more immunogenic as opposed to the humanized ocrelizumab (3, 5, 9, 17).

A more detailed analysis of the demographic and clinical features of anti-CD20 induced colitis can be found in an article published by Tolaymat et al. (6). No incidence of this adverse event has been reported so far. As of February 2024, more than 300.000 patients have been reported to be treated with ocrelizumab.^1^ Adding our cases to the already reported ones, this would result in 54 cases of ocrelizumab-induced colitis for 300.000 treated patients (0,018%).

Bezzio et al. (48) named shared susceptibility genes between IBD and MS as a proof of a,common genetic background” and underlined that Th17 cells are an important participant in both diseases, producing pro-inflammatory cytokines (IL-17, IL-22), with high IL-17 levels being found in both pathologies. Breg cell dysfunction and numerical reduction was also emphasized in UC, with reduced IL-10 levels as a result, leading to uncontrolled inflammatory responses (19, 49). As such, ocrelizumab-induced colitis is believed to be a consequence of B cell depletion, particularly Bregs, with subsequent reduction of IL-10 secretion, which leads to uninhibited Th1 cell activity and predisposes the patient to diminished anti-inflammatory responses (6–15, 25).

The contribution of gut microbiome alteration and mucosal dysfunction in MS patients (50) to B cell depletion caused by anti-CD20 therapy, as well as common genetic and environmental risk factors for MS and IBD, may have a significant impact as co-factors in a more complex association of pathophysiological mechanisms (25). Histological studies revealed a modified gastrointestinal mucosa in MS patients (“leaky gut”) - an increased intestinal permeability which allows translocation of immunogenic molecules that can trigger an autoimmune response (51–54).

While some authors (24) question if ocrelizumab only acts as a trigger in exposing complex immunological changes that lead to gastrointestinal autoimmune diseases in pwMS (as opposed to a directly induced drug side-effect), numerous reports from patients treated with ocrelizumab and rituximab plead in favor of a class-related adverse event, which was also emphasized by Tolaymat et al. (6). This is further supported by a cohort study on cancer patients treated with rituximab as part of their chemotherapy regimen, who developed gastrointestinal toxicities and colitis (47).

### Proposed diagnostic criteria for ocrelizumab-induced colitis – real-world evidence of their applicability

An article by Quesada-Simo published in 2023 proposed diagnostic criteria for ocrelizumab-induced colitis (see above – Methods). A diagnosis of ocrelizumab-induced colitis can be established if at least three major criteria and two minor ones are fulfilled (16). However, attending physicians must bear in mind these observations:

1. In several circumstances, overlapping infections can conceal the drug-induced adverse event. Persistence of symptoms despite adequate antimicrobial treatment and a favorable response to anti-inflammatory therapies should lead the clinician to reconsider the diagnosis. A high index of suspicion is necessary to avoid a delayed recognition of drug-induced colitis.
2. Some cases may not entirely meet these requirements and yet a causal association can exist, if considering the temporal relationship to ocrelizumab exposure and other contributory or confounding factors, as per the assessment made by Kim et al. (25).

No set of diagnostic criteria can be perfect, and one must remember that no data is available regarding the sensitivity and specificity for the established criteria for ocrelizumab-induced colitis due to the very low number of reported cases.

When suspecting ocrelizumab-induced colitis, infectious causes should be investigated (16). Comprehensive infectious screening should include bacterial causes (e.g., *Salmonella*, *Shigella*, *Campylobacter*, *E. coli*, *C. difficile*), viral (CMV, HSV 1/2) and, if needed, for parasitic and fungal pathogens. Still, as highlighted by Challa et al. (26) and by our second case, infections can be superposed to the underlying anti-CD20 mediated event.

A differential diagnosis should also be considered with IBD [Ulcerative colitis/Chron’s Disease (UC/CD)], as associations between MS and IBD have been reported independent of ocrelizumab therapy. While clinical presentation may overlap, further serological, endoscopic and histopathological findings will help differentiate these entities. However, the following points must be taken into consideration:

- IBD has been described as a consequence of anti-CD20 therapies in several case reports (9, 10, 15, 31), however the diagnosis was mainly established based on histopathological aspect, not on the disease course due to heterogenous and mostly short follow-up periods.
- While these are two distinct entities and diagnostic criteria should help distinguish them (16, 25), certain forms of Ocrelizumab-induced colitis might present histopathological similarities to IBD and should rather be considered IBD-like (16, 25). In such cases, they should be classified as, “anti-CD20-induced IBD-like colitis” as a subtype of drug-induced colitis rather than IBD.

### Risk factors for ocrelizumab-induced colitis

No risk factors for ocrelizumab-induced colitis have been clearly established so far (16), but the following have been hypothesized:

- Genetic factors: genetic susceptibility risk (50, 55) (assessment of family history (16)), which can be extrapolated from the already established genetic correlation between MS and IBD, with genes such as IFNG (regulation of Th1 and Th2 cytokines), PTGER4 (via prostaglandin E, which regulates cytokines), CXCR6 (expressed in CD8+ T cells) or GPR25 being investigated by Yang et al. (55).
- Immunologic factors: lymphopenia (16), hypogammaglobulinemia (3, 5, 16, 17, 56).
- Drug-related factors: previous use of DMTs (13, 16), number of ocrelizumab administrations (26), other immunosuppressive drugs (16).

While one paper reported the degree of lymphopenia as being correlated with colitis (16), Mallepally et al. (47) state that there is no correlation between immunosuppression and occurrence of colitis, and it cannot be used as an means for identifying patients at risk for developing anti-CD20-induced colitis.

MS and IBD have common risk factors such as smoking, vitamin D deficiency, infections, cold climate or higher socioeconomic status (6, 16, 48, 57–59). No association has been previously investigated between these and anti-CD20 induced colitis and further studies will be necessary.

A recent study suggests that colitis may occur in a dose-dependent manner, with more severe cases being observed after prolonged ocrelizumab treatment (26). This observation is challenged by case reports which describe severe intestinal consequences after a single dose of ocrelizumab requiring surgical intervention or onset of digestive symptoms shortly after exposure to anti-CD20 therapy (6, 8, 11, 12, 31).

For our 3 reported cases, we observed previous use of DMTs (1 out of 3 patients) and hypogammaglobulinemia (2 out of 3 patients) as potential risk factors. Symptoms onset ranged from 1 to 6 infusions, however an increase in the severity of symptoms was observed with each infusion, with the most severe form occurring to the patient with 6 infusions (Appendix 1).

### Treatment of ocrelizumab-induced colitis. Available evidence and the experience of our MS center

The management of these patients is heterogenous in case reports (7–15, 27, 60). Starting from general treatment recommendations for IBD and based adapting to the proposed etiology of ocrelizumab-induced colitis, some key points can be made:

#### Discontinuation of ocrelizumab

If colitis is considered to be a drug-induced event, discontinuing the causative factor is an appropriate measure. Most of the case reports used this approach, with favorable clinical and endoscopic evolution at follow-up (6, 16, 26). Resolutions is expected once the predisposing agent has been stopped, as opposed to IBD and its relapsing–remitting nature. Nonetheless, in some situations, ocrelizumab was continued, such as when mild digestive manifestations (26) could be managed conservatively or due to lack of alternative therapies (10). The possibility of an adapted dosing regimen (30, 31) was also described. Regarding a reattempt of ocrelizumab treatment, there is limited and inconclusive data, as most reports opted for a permanent discontinuation, given the severity of the gastrointestinal adverse event (27). Initiation of targeted treatment for colitis, whether medical or surgical, may act as a confounding factor for properly evaluating this matter (27).

#### Biologic therapies and DMT switch

Therapeutic approaches for patients who need a DMT switch that have been reported are the use of natalizumab, ozanimod, or dual biologic therapy (DBT) with ustekinumab or vedolizumab combined with an MS-specific DMT with no gastrointestinal adverse reactions or contraindications in IBD (7–15). Natalizumab has been proposed and used for its mechanism as an integrin inhibitor (α4ß1 and α4ß7), which blocks the passage of lymphocytes at the gastrointestinal level and in the CNS (6, 8, 15, 16). Ozanimod, a S1P receptor modulator, has been approved for treating UC and favorable effects have been reported when used for CD (16). Nevertheless, data is lacking for the use of ozanimod for this pathology and this treatment choice may not be as potent as needed for a highly-active form of MS (27). Keeping the dual benefits of these therapies in mind, they would represent ideal options for both disease entities. One case report suggests cladribine for the DMT switch, yet this was not further investigated (6). This could be an option for patients with a positive JCV index who should receive high-efficacy therapy. Another options for this circumstance may be ofatumumab or even alemtuzumab, as stated by Viti et al. (27). However, the authors of this paper raised potential caution regarding these therapies: ofatumumab has been reported to have a higher risk of colitis among anti-CD20 therapies, albeit without real-world data available, while alemtuzumab may exhibit pro-immune properties which are not desired when treating an immune-mediated pathology (27).

Regarding ustekinumab and vedolizumab, these are mainly used in IBD, but case reports show favorable effects for these therapies in anti-CD20 induced colitis. While vedolizumab was chosen for its mechanism, acting as an α4ß7 integrin inhibitor (a mechanism similar to natalizumab, but without effects at the CNS level), ustekinumab was selected as a salvage therapy, with no proven effect as a MS-DMT (8, 16, 43).

The timing of initiation of a new DMT is problematic. Quesada-Simó et al. (16) suggested a “reasonable wash-out period,” remission of the inflammatory syndrome and B cell repopulation. The depletion of B cells after administration of ocrelizumab occurs in 2 weeks and B cell levels return to their initial level after a median of 72 weeks after the last infusion (1, 3, 61). The prolonged steroid tapering and depletion of B cells after anti-CD20 therapy may offer a protection against MS relapses. However, withholding DMTs for as long as the reported interval to normalization of B cells would be too long for patients deemed eligible for high-efficacy DMT. We suggest periodic clinical and biological assessments (including routine blood counts, immunoglobulin levels and CD19+/CD20+ B cell subtypes), at 3-months interval, to determine the optimal time to start a new DMT, balancing the need for remission of the gastrointestinal pathology with avoiding the risk of MS relapses and progression.

#### Supportive care (fluid therapy, dietary changes and symptomatic treatment)

These are the mainstays in the management of acute colitis regardless of its etiology. Patients should be monitored for malnourishment and, if necessary, repletion with albumin may be considered (62). Guidelines for an appropriate diet in the acute phase are available (63–66). Whether enteral nutrition should be initiated depends on the clinical status of the patient. Regarding symptomatic treatment, there are reports of using loperamide (16, 26), yet this drug is not indicated in case of bloody diarrhea. In our case series, spasmolytic drugs (trimebutine, alverine, drotaverine) were successfully used for relief of abdominal cramps.

#### Corticosteroids

Various forms of administration are reported, such as intravenous with/ without an oral tapering, oral, or corticosteroid enemas (6, 16). Hydrocortisone i.v., methylprednisolone i.v., oral prednisone/ prednisolone or budesonide were all used for patients anti-CD20 induced colitis. Even if not reported in antiCD20-induced colitis, the available IBD guidelines (63–66) and other authors recommend considering the use of topical steroids for avoidance of systemic adverse reactions or as an add-on treatment for IBD (67). For the first two cases from our cohort, the route of administration was adapted to the severity of colitis. As such, an intravenous course of steroid therapy followed by extended oral tapering was administered to our first patient, who had severe clinical manifestations and extensive colonic ulcerations, while our second case was managed successfully with combined oral therapy (steroids +5-ASA).

#### Aminosalicylates

Mesalamine/ mesalazine/ 5-aminosalicylic acid (5-ASA) administered orally are an important pillar in the treatment of IBD and were also used in some of the case reports of anti-CD20 induced colitis (16, 33). Aminosalicylates and corticosteroids are used for their anti-inflammatory and immunosuppressive properties (68). In IBD guidelines, 5-ASA is used for mild to moderately active forms of disease (62). Rectal forms are also available and some case reports associated sulfasalazine (26). Mesalamine orally was used for both of our first two patients in the acute phase of the disease.

#### Surgical treatment

Severe, unresponsive cases of anti-CD20 induced colitis required extensive surgical approach, in extreme cases even total colectomy (6, 13, 16). Decisions of this extent must be discussed on a case-by-case basis within the multidisciplinary team.

#### Active infections

Superposed infections lead to an even more challenging management (26). Infectious screening and correctly establishing the causality are mandatory, and antibiotic, antiviral or antifungal therapy should be applied if patients have positive infectious tests, as infections will further aggravate the course of the disease.

Available treatment strategies for ocrelizumab-induced colitis as applied for our subjects can be found in Appendix 3.

## Conclusion

Ocrelizumab-induced colitis is a rare, but severe adverse event. Its incidence may be higher than expected, reaching 1,95% in our cohort of MS patients, compared to the estimated general incidence of 0,018%. Screening ocrelizumab-treated patients for digestive symptoms could help detect such cases in an incipient stage. It is encouraged to further report cases of ocrelizumab-induced colitis in order to broaden our understanding of this side effect.

## Data Availability

The original contributions presented in the study are included in the article/Supplementary material, further inquiries can be directed to the corresponding author/s.

## Footnotes

^1^ https://assets.roche.com/f/176330/x/01089f380d/roche-launches-ocrevus_-press-release-final.pdf
