# Supplementary material for: Ocrelizumab-induced colitis—critical review and case series from a Romanian cohort of MS patients
Source: Front Neurol. 2025 Feb 5;16:1530438. doi: 10.3389/fneur.2025.1530438 (PMC11835689; doi:10.3389/fneur.2025.1530438)
Supplement: Supplementary file 1 [file Table_1.DOCX]

**Appendix 1 - Clinical data of the reported cases**

| **Main characteristics** | **Case 1** | **Case 2** | **Case 3** |
| --- | --- | --- | --- |
| **Gender** | female | male | female |
| **Age** | 35 | 26 | 37 |
| **Environment** | urban | urban | urban |
| **Family history of IBD** | no | no | no |
| **Smoking status** | ex-smoker | non-smoker | current smoker |
| **Year of MS diagnosis** | 2021 | 2019 | 2022 |
| **MS form** | relapsing-remitting | relapsing-remitting | relapsing-remitting |
| **EDSS at onset** | 2.5 | 1 | 2 |
| **Current EDSS** | 3 | 1.5 | 1.5 |
| **Year of ocrelizumab initiation** | 2021 | 2022 | 2022 |
| **Previous DMT** | no | yes | no |
| **Onset of digestive symptoms** | 2023 | 2022 | 2023 |
| **Number of ocrelizumab infusions prior to digestive symptoms** | 6 | 1 | 3 |
| **Gastrointestinal manifestations** | abdominal cramps  urgent defecation  loose, watery, sometimes bloody stools  weight loss | abdominal pain and cramps  loose stools | watery stools, sometimes accompanied by blood streaks |
| **Laboratory results** | increased ESR and CRP  elevated fecal calprotectin  negative infectious screening | elevated fecal calprotectin  superimposed bacterial infections in the course of the disease (C. difficile; C. jejuni) | no biological abnormalities |
| **Decreased Ig levels** | yes | yes | not performed |
| **Colonoscopy** | multiple profound colonic ulcerations | erythematous areas, microerosions, edema and erythema of various segments of the colonic mucosa | no significant macroscopic anomalies |
| **Other investigations** | abdominal CT scan – minimal colonic edema | not performed | not performed |
| **Histopathological report** | focal active colitis | chronic active non-granulomatous colitis | no signs of inflammation or dysplasia |
| **Immunohistochemistry staining** | complete depletion of CD19+ and CD20+ B cells  positive for CD3+ T cells and CD79+ B cells*  negative for CMV | near-total depletion of CD20+ B cells  abundant CD79+ B cells  frequent CD3+ T cells  negative for HSV1/2  negative for CMV | absence of CD20+ B cells  CD79+ B cells present  CD3+ T cells present  negative for CMV |

C. difficile: Clostridium difficile, C. jejuni: Campylobacter jejuni, CMV: Cytomegalovirus, CRP: C-reactive protein, CT: Computed Tomography, DMT: disease modifying therapy, EDSS: Expanded Disability Status Scale, ESR: erythrocyte sedimentation rate, HSV: herpes simplex virus, IBD: inflammatory bowel disease, Ig: immunoglobulin, MS: multiple sclerosis

*CD79+ B cells, though a marker of the complete lineage of B cells, may be interpreted as an expression of abundance of plasmocytes in this context, while infiltrates of CD3+ T cells are an expression of the active immune response
