# Supplementary material for: Ocrelizumab-induced colitis—critical review and case series from a Romanian cohort of MS patients
Source: Front Neurol. 2025 Feb 5;16:1530438. doi: 10.3389/fneur.2025.1530438 (PMC11835689; doi:10.3389/fneur.2025.1530438)
Supplement: Supplementary file 2 [file Table_2.DOCX]

**Appendix 2 – Proposed criteria for the diagnosis of ocrelizumab-induced colitis^16^ applied to our subjects**

| **Diagnostic criteria** | **Case 1** | **Case 2** | **Case 3** |
| --- | --- | --- | --- |
| **Major criteria** | | | |
| Exposure to an anti-CD20 drug in the previous year | √ | √ | √ |
| Compatible symptoms that may include fever, abdominal pain, watery or muco-bloody diarrhea | √ | √ | √ |
| CD20+ cell depletion on GI biopsy | √ | √ | √ |
| Lymphoplasmocytic infiltrate (CD3+ and CD79+ plasma cells) in the lamina propria | √ | √ | - |
| Clinical/ endoscopic recovery after drug withdrawal and CD20+ cell recovery in intestinal mucosa | √ | √ | - |
| **Minor criteria** | | | |
| Elevation of acute inflammation biomarkers in laboratory tests (CPR, ESR, lymphocytosis) | √ | √ | - |
| Compatible endoscopic findings (mucosal erythema with edema and patchy ulcer/ erosions with predominant involvement of the ileum and proximal colon; tendency to spare stomach and duodenum) | √ | √ | - |
| Chronic active inflammation with cryptitis, goblet cell reduction, and superficial ulcers with areas of spared mucosa | +/- | √ | - |
| Good response to glucocorticoid therapy | √ | √ | - |
| **Absence of other possible etiologies** | | | |
| Normal neutrophil count | √ | +/-* | √ |
| Absence of infectious causes (CMV, Salmonella, Shigella, Campylobacter, E. coli, C. difficile) | √ | +/-* | √ |
| Other comorbidities that could justify the pathology | - | - | - |
| Other drugs/ toxics that could justify the pathology | - | - | - |

C. difficile: Clostridium difficile, CMV: Cytomegalovirus, CRP: C-reactive protein, E.coli: Escherichia coli, ESR: erythrocyte sedimentation rate, GI: gastrointestinal

The diagnosis of ocrelizumab-induced colitis is made if at least three major criteria and two minor ones are accomplished.^16^

* The infectious screening was positive after the superimposed infections with C. difficile and C. jejuni occurred
