# Supplementary material for: Ocrelizumab-induced colitis—critical review and case series from a Romanian cohort of MS patients
Source: Front Neurol. 2025 Feb 5;16:1530438. doi: 10.3389/fneur.2025.1530438 (PMC11835689; doi:10.3389/fneur.2025.1530438)
Supplement: Supplementary file 3 [file Table_3.DOCX]

**Appendix 3 – Available treatment strategies for ocrelizumab-induced colitis^1-10^ as applied for our subjects**

| **Treatment** | **Case 1** | **Case 2** | **Case 3** |
| --- | --- | --- | --- |
| **Fluid therapy** | yes | yes | no |
| **Dietary changes** | yes | yes | no |
| **Symptomatic treatment** | spasmolytics  analgesics | spasmolytics | antisecretory drugs |
| **Antibiotic/ antiviral treatment** | amoxicillin + clavulanic acid* | fidaxomicin, vancomycin, azithromycin* | no |
| **Corticosteroids** | yes – IVMP and oral tapering | yes – oral prednisone | no |
| **5-ASA** | yes | yes | no |
| **Biologic therapies (eg. ustekinumab, vedolizumab)** | no | no | no |
| **Surgery** | no | no | no |
| **Ocrelizumab discontinuation** | yes | yes | under evaluation |
| **DMT switch** | natalizumab | under evaluation for cladribine | no |
| **Outcome** | clinical resolution in 1-2 months, favorable endoscopic aspect at 3 months | slow clinical resolution due to lack of adherence | under evaluation |

5-ASA: 5-aminosalicylates, DMT: disease modifying therapies, IVMP: intravenous methylprednisolone

* Prior to admission in our hospital

1. Tuqan W, Siddiqi F, Ray A. S1766 Ocrelizumab-induced colitis: A case report. Am J Gastroenterol. 2020;115:S913.
2. Shah, J., Al-Taaee, A., Deepak, P., Gremida, A., 2022. Severe ocrelizumab-induced enterocolitis treated successfully with ustekinumab. ACG Case Rep. J. 9 (1), e00742.
3. Barnes A, Hofmann D, Hall LA, Klebe S, Mountifield R. Ocrelizumab-induced inflammatory bowel disease-like illness characterized by esophagitis and colitis. Ann Gastroenterol. 2021;34(3):447-448. doi: 10.20524/aog.2021.0582. Epub 2021 Jan 27. PMID: 33948072; PMCID: PMC8079880
4. Carballo-Folgoso L, Celada-Sendino M, Castaño-García A, Oliva Nacarino P, Morales Del Burgo P, Pérez-Martínez I, de Francisco R, Riestra S. Crohn's disease induced by ocrelizumab in a patient with multiple sclerosis. Rev Esp Enferm Dig. 2022 Dec;114(12):766-767. doi: 10.17235/reed.2022.9152/2022. PMID: 36177822
5. Akram, A., Valasek, M., Patel, D., 2020. P096 De novo colitis after ocrelizumab therapy. Gastroenterology 158 (3), S1–S2.
6. Sunjaya DB, Taborda C, Obeng R, Dhere T. First Case of Refractory Colitis Caused by Ocrelizumab. Inflamm Bowel Dis. 2020 May 12;26(6):e49. doi: 10.1093/ibd/izaa057. PMID: 32198886
7. Lee HH, Sritharan N, Bermingham D, Strey G. Ocrelizumab-Induced Severe Colitis. Case Rep Gastrointest Med. 2020 Dec 7;2020:8858378. doi: 10.1155/2020/8858378. PMID: 33354373; PMCID: PMC7737437
8. Elands S , Lemmers A, Delhaye M, Franchimont D, Liefferinckx C, Pezzullo M and Perrotta G. Secondary Autoimmune Diseases Following Ocrelizumab Therapy for Multiple Sclerosis, www.charcot-ms.org/files/Annual-Meetings/28/Posters-2020/pdf-format/43_ECF2020_Poster_TR_Elands_S.pdf
9. Au M, Mitrev N, Leong RW, Kariyawasam V. Dual biologic therapy with ocrelizumab for multiple sclerosis and vedolizumab for Crohn's disease: A case report and review of literature. World J Clin Cases. 2022 Mar 16;10(8):2569-2576. doi: 10.12998/wjcc.v10.i8.2569. PMID: 35434082; PMCID: PMC8968582
10. Quesada-Simó A, Garrido-Marín A, Nos P, Gil-Perotín S. Impact of Anti-CD20 therapies on the immune homeostasis of gastrointestinal mucosa and their relationship with de novo intestinal bowel disease in multiple sclerosis: a review. Front Pharmacol. 2023 May 30;14:1186016. doi: 10.3389/fphar.2023.1186016. PMID: 37324473; PMCID: PMC10263191
